# Forced Social Isolation and Mental Health: A Study on 1,006 Italians Under COVID-19 Lockdown

**DOI:** 10.3389/fpsyg.2021.663799

**Published:** 2021-05-21

**Authors:** Luca Pancani, Marco Marinucci, Nicolas Aureli, Paolo Riva

**Affiliations:** Department of Psychology, University of Milano–Bicocca, Milan, Italy

**Keywords:** COVID-19, social isolation, offline contacts, online contacts, mental health, space adequacy, virus local spread

## Abstract

Most countries have been struggling with the spread of the COVID-19 pandemic imposing social isolation on their citizens. However, this measure carried risks for people's mental health. This study evaluated the psychological repercussions of objective isolation in 1,006 Italians during the first, especially strict, lockdown in spring 2020. Although varying for the regional spread-rate of the contagion, results showed that the longer the isolation and the less adequate the physical space where people were isolated, the worse the mental health (e.g., depression). Offline social contacts buffered the association between social isolation and mental health. However, when offline contacts were limited, online contacts seemed crucial in protecting mental health. The findings inform about the potential downsides of the massive social isolation imposed by COVID-19 spread, highlighting possible risk factors and resources to account for implementing such isolation measures. Specifically, besides some known factors such as physical space availability, the local contagion rate is critical in moderating the link between social isolation and mental health issues, supporting national policies implementing regional tiers of restriction severity.

## Introduction

Since early 2020, the COVID-19 pandemic caused about 4 billion people to be confined to their homes. Physical distancing has been adopted by most of the affected countries, including Italy, the first western country hit by the virus. The restrictions followed the health situation trend, for which a series of lockdowns alternated with less restrictive phases. However, particularly in the Italian context, no lockdown was comparable with the first one regarding the strictness of the measures taken to confine citizens to their homes.

The rapid spread of the COVID-19 forced the Italian government to apply drastic measures to tackle the contagion. The government enacted a decree at the beginning of March 2020, imposing a lockdown on the whole country, aimed at preventing the coronavirus from spreading in areas where the contagion was already extremely critical (e.g., the Lombardy region) and in those with only a few cases. Schools and universities closed for the entire semester, all the non-essential activities (e.g., bars and restaurants) were closed, and public gatherings were forbidden. Most people were forced to stay at home when the government prohibited people from leaving their houses unless for proven necessity, otherwise meeting harsh sanctions. Even city parks were closed, and outdoor physical activity was banned. Local police passed through the city streets, reiterating the need for citizens to stay at home over the loudspeakers. The failure to comply with the dispositions was punishable with fines or even imprisonment (Lattanzi, 2019). In this sense, the characteristics of the first Italian lockdown were unique for western countries. It was totally unexpected and implemented in an extremely harsh and unprecedented way compared with both the first lockdowns of other western countries and the following lockdowns enacted in Italy.

Most importantly, such severe restrictions were applied uniformly across the entire country, regardless of the contagion's local spread (high vs. low). For instance, while in the Lombardy region, the spread of the infection was very high, with about 40% of all the Italian positive cases registered in March 2020; in the Calabria region, it was almost non-existent, representing only 0.6% of all the positive cases (Dipartimento della Protezione Civile, 2020). Despite this, the first lockdown characteristics in terms of social isolation were uniform throughout the national territory.

Despite the efforts of many scholars during the last few months, the research on the negative psychological repercussions of social isolation is still underway and many questions remain unanswered. The present study focused on the first phase of the COVID-19 pandemic in Italy, investigating the link between forced isolation and mental health by accounting for the role of the regional contagion rate, offline and online social contacts, and the adequacy of living space.

### The Impact of Social and Physical Isolation on Mental Health

Social isolation refers to an objective physical separation from others and is different from loneliness, which is a subjective feeling of disconnectedness (Cacioppo and Patrick, 2008). It is known that brief forms of social disconnections can induce negative emotions (such as anger and sadness), and decrease satisfaction of basic psychological needs (e.g., self-esteem) and cognitive abilities. On the other side, prolonged social disconnection experiences have been linked with an increased risk of depression, suicidal thoughts, and risk of early mortality (Baumeister and Leary, 1995; Holt-Lunstad et al., 2010). The Temporal Need-Threat Model (Williams, 2009) suggests that people exposed to long-lasting instances of social exclusion—defined as the experience of being kept apart from others physically or emotionally (Riva and Eck, 2016)—enter a stage of psychological resignation, characterized by feelings of depression, alienation, unworthiness, and helplessness. Other theoretical models associated social withdrawal behaviors with prolonged rejection (Smart Richman and Leary, 2009). However, such exclusion-related implications for mental health have been found predominantly either in persistently marginalized social groups—such as immigrants (Marinucci and Riva, 2020a)—or in individuals with ostracism experiences that could last for years (Zadro, 2004). Moreover, the literature on loneliness has highlighted a significant relationship with mental health (Cacioppo and Patrick, 2008). However, besides being a subjective perception, loneliness refers to a stable individual disposition, hence a construct that again persists over time. Thus, investigating the effects of loneliness on psychological well-being differs much from addressing whether forcing the general population to remain isolated for a limited period (a few days or weeks) could produce a drop in mental health levels.

The available literature highlights that the quantity and quality of face-to-face social connections could influence the psychological health of individuals exposed to persistent conditions of exclusion (Baumeister and Leary, 1995). For instance, a study in the prison setting showed that inmates attending in-person group meetings presented significantly better mental health than prisoners who did not join the group sessions (Aureli et al., 2020). Similarly, face-to-face interactions with native people protected immigrants' psychological health from the harm of social exclusion (Marinucci and Riva, 2020b). Besides in-person relationships, also online social interactions via information and communication technologies (e.g., social networking sites) could protect from the mental health impact of persistent exclusion and isolation. Waytz and Gray (2018) emphasized that digital technologies could foster sociability and human relatedness when objective constraints impede face-to-face interactions. Concerning the COVID-19 pandemic, a study conducted in Italy during the first lockdown in 2020 showed that using technologies to relate with other people (from multiplayer videogames to leisure meetings and work-related video calls) was positively associated with psychological well-being via perceived social support (Gabbiadini et al., 2020). Hence, the research suggests that the quantity and quality of face-to-face and online social interactions could buffer from the harm of prolonged conditions of social isolation, as in COVID-19 lockdown.

### Isolation Length, Virus Local Spread, and Adequacy of the Living Space

The first Italian lockdown occurred between March 9 and May 3, 2020, offering the possibility to explore whether the effects of social disconnection on mental health may occur even for relatively short periods. Moreover, the lockdown permitted the empirical investigation of the effects of the following objective conditions on the general population: the length of the social isolation period and the possible moderating effect of the pandemic's local severity.

The length of the isolation period, measured by assessing the number of days since the beginning of the lockdown, would allow controlling for confounding correlation between the individual subjective perception of social disconnectedness (e.g., loneliness) and mental health. Previous research on people under quarantine showed that such an experience could have significant downsides (Barbisch et al., 2015; Rubin and Wessely, 2020). In 2020, many studies focused on the impact of COVID-19 quarantine on mental health, especially in China, the site of the first outbreak of the virus. These studies suggested detrimental effects of the lockdown on various indicators of mental health, such as life satisfaction, psychological distress, and insomnia (Torales et al., 2020; Wang H. et al., 2020; Zhang and Ma, 2020; Zhang et al., 2020), and similar results were also obtained on the Italian population (Gualano et al., 2020; Rossi et al., 2020). A Spanish study on helpline psychological counselors observed that the lockdown generated/aggravated people's family and mental health problems, increasing their anxiety and feeling of loneliness (Hervalejo et al., 2020). Similarly, a multi-country study showed a wide range of psychological consequences of home confinement, including poor sleep quality and unhealthy lifestyle behaviors, such as physical and social inactivity (Ammar et al., 2020). However, no measures of lockdown length were taken into account. Wang C. et al. (2020) made an effort in this direction, conducting a longitudinal study in China with two waves, one during the initial outbreak and the second 4 weeks later. Although the researchers identified some protective factors for mental health during the lockdown (e.g., confidence in doctors, risk perception), they did not observe any worsening in mental health: psychological distress did not change between the first and the second wave, whereas post-traumatic disorder decreased over time, even though this reduction was not clinically relevant. Conversely, an Italian longitudinal study conducted at the beginning and the end of the first lockdown showed small to medium worsening in the levels of depression and stress, but not in anxiety (Roma et al., 2020).

A recent systematic review (Brooks et al., 2020) revealed that quarantined people reported various psychological issues, such as acute stress symptoms, anxiety, insomnia, and emotional exhaustion. However, the review included studies with heterogeneous samples, such as individuals quarantined for being in contact with infected people, individuals only living in outbreak sites, and nurses and physicians directly involved in tackling the infection. Moreover, only three studies out of 24 considered the quarantine length as a predictor of mental health. Thus, the review does not account for the psychological impact of the lockdown length imposed on the general population. Indeed, the authors highlighted that the length of the quarantine and the disruption of social connections might be responsible for the quarantine's negative psychological repercussions, calling for further studies directly assessing the role of these potential mechanisms.

Beyond the length of social isolation, the virus local spread might represent a key factor in determining people's mental health issues during a lockdown. According to WHO, particular attention should be devoted to mental health in areas strongly affected by COVID-19 (World Health Organization, 2020). People living in high-contagion areas might perceive and experience the threat differently from those living in low-contagion areas. However, the literature on the relationship between the local spread of the virus and people's psychological well-being is limited and provided mixed results. A recent study on the COVID-19 pandemic showed that anxiety (but not depression) was more prevalent in Hubei province (China's worst-hit province) than in other areas of the country (Gao et al., 2020). Similarly, a large Italian study conducted during the first lockdown showed that people living in the south of the country were more likely to experience mental health issues (e.g., depression, anxiety, insomnia) than those living in the north (Rossi et al., 2020). Although the authors did not measure the actual contagion rate, southern Italy had a lower local spread than northern, suggesting a link between contagion rate and mental health. Opposite results were obtained by Ahmed et al. (2020), who observed severe depression symptoms (but not anxiety) in Hubei inhabitants more than twice as frequently as in people living in other Chinese areas.

Finally, the restrictions confining people to stay home for several consecutive days may represent an additional risk factor for mental health. Indeed, the living space's characteristics, including its size, luminosity, and the possibility of privacy, may crucially moderate people's experience of isolation (WHO/Europe, 2007). Literature suggests that an inadequate home environment (e.g., tiny apartments, low levels of natural light) can lead to both physical (e.g., respiratory morbidity) and psychological (e.g., negative feelings) consequences, compromising psychological well-being (Jones-Rounds et al., 2014). Although spending time outside might help people to cope with inadequate living spaces, the lockdown limits this opportunity and, therefore, inappropriate dwellings may worsen mental health.

### The Present Study

The present study aimed to test the relationship between the length of forced isolation and the adequacy of living space on mental health during the first wave of the COVID-19 pandemic in Italy, considering the key role of differences in the local spread of the virus. We focused on mental health outcomes (e.g., depression) that previous research linked with prolonged exclusion and isolation experiences (Williams, 2009). Specifically, we tested whether

1. The longer the forced isolation (measured objectively as the number of days between the beginning of the lockdown and the day of completion of the survey), the more negative mental health outcomes.
2. The number of offline contacts available could mediate the relationship between forced isolation and mental health. Specifically, fewer face-to-face relationships due to forced isolation could worsen mental health. Differently, online contacts could buffer the negative relationship between days of isolation and mental health.
3. Being confined in inadequate physical spaces would be associated with worse mental health.

All the associations mentioned previously were tested separately for people living in high- vs. low-contagion areas to account for the high variability in the infection rate in Italy during the first wave of the pandemic. Indeed, the gap between the harshness of the restrictions and the actual contagion spread could constitute an additional source of burden in an already distressful condition. Specifically, we split the sample into two sub-samples according to the official data about the regional level of contagion (Dipartimento della Protezione Civile, 2020), comparing the associations among the focal variables between the two groups.

## Materials and Methods

### Participants and Procedure

The present study was approved by the Ethics Committee of the Department of Psychology at the University of Milano–Bicocca (approval number: RM-2020-263). Written consent was obtained by all the participants included in the data analysis. The survey was set up using Qualtrics (2020). The study was advertised on various social media with a brief post explaining our general aim (i.e., investigate habits and psychological well-being during the COVID-19 pandemic), trying to cover the whole country by posting on pages of different regions. The post included the link to the online survey. Once clicked on the link, participants were initially presented with the information sheet and consent form. Data were collected after the enactment of the lockdown by the Italian government (March 9), specifically between March 12 and 27, 2020. All data and the codebook are available at https://osf.io/xb8yj/?view_only=966abafccc844b99924da85be3f76272.

Overall, a convenience sample of 2470 persons accessed the online study. However, 328 participants only opened the link, and 22 did not give their consent; thus, they did not fill in any questions, reducing the sample size to 2120. Among these participants, 783 did not complete one or more independent variables (i.e., gender, age, space adequacy), further reducing the sample to 1337 individuals. Then, metadata on location were not automatically collected for 91 participants, whereas 11 participants compiled the survey abroad (i.e., outside Italy); thus, the sample was further reduced to 1,235 cases.

A final reduction was made based on the answers to offline and online contacts. Specifically, we asked participants to list up to 10 offline and online contacts (i.e., 20 contacts at the most) and to rate the closeness with each of them during the previous week (for further details, see section Offline and Online Social Contacts). For both offline and online contacts, cases were excluded if at least one of the following conditions was met: (1) at least one entry of the list clearly referred to multiple persons (e.g., “relatives,” “friends,” “colleagues”); (2) at least one entry that missed either the reference to a specific person or closeness rating; (3) all the entries were left blank and the participant declared it was not done on purpose. Forty-nine participants did not meet at least one of the aforementioned criteria for offline contacts, 105 for online contacts, and 75 for both offline and online contacts. Thus, the final sample on which the analyses were conducted consisted of 1,006 participants.

The sample size (*N* = 1006) was considered appropriate for the planned analysis, given that it largely exceeded the recommendation of 20 cases for each estimated parameter in a structural equation model (Kline, 2015). The sample was unbalanced for gender, including 807 females (80.2%), with an age range between 18 and 75 years, *M* = 29.57, SD = 10.89, and consisted of 984 participants of Italian nationality (97.8%). Concerning occupational status, 581 participants were employed (57.8%). Concerning education, 508 participants (50.5%) had a bachelor's degree or a higher education level, 463 (46.0%) a high school degree, and 35 (3.5%) a lower education level. The number of people living with participants ranged between 0 and 9, *M* = 2.22, SD = 1.31 (two participants did not answer this question).

### Materials

The Qualtrics platform automatically gathered the date of survey completion and the geographical area in which the survey was compiled. The date of completion of the survey was used to compute the number of days since the official lockdown, which was considered the main proxy for social isolation length. Thus, the first day of data collection (March 12) was coded 4 (4 days from the beginning of the lockdown on March 9), and the last day (March 27) was coded 20. The location was used to objectively assess the level of contagion in the region where participants lived on the date of survey completion. Specifically, for each participant, we computed the daily percentage of regional COVID-19-positive individuals over the total infected in the Italian population, based on the official data of the Italian public safety department (Dipartimento della Protezione Civile, 2020).

Beyond sociodemographic information (i.e., gender, age, nationality, occupation, education, number of people living with participants), the survey included the following measures.

#### Mental Health Issues

Based on Williams' theory 2009, mental health issues were evaluated by measuring the four long-term negative consequences of social isolation, namely depression, unworthiness, alienation, and helplessness. Following the procedure of previous research (Riva et al., 2017; Marinucci and Riva, 2020a), we selected a subset of five items from psychometrically valid scales measuring the four constructs to keep the measure as short as possible. Item selection was primarily based on items loading (i.e., the highest, the better) and avoiding overlaps with items measuring other constructs. Participants were asked to indicate how often the events reported by the 20 items occurred during the last week, from 1 (not at all) to 7 (always). Items measuring depression derived from the Depression Anxiety and Stress Scales (α = 0.89; sample item: “I felt down-hearted and blue”; Henry and Crawford, 2005). Items measuring unworthiness derived from the Rosenberg Self-Esteem Scale (α = 0.78; sample item: “At times, I thought I am no good at all”; Rosenberg, 1965). Items measuring alienation derived from the Social Connectedness Scale (α = 0.82; sample item: “I felt disconnected from the world around me”; Lee and Robbins, 1995). Items measuring helplessness derived from the Beck Hopelessness Scale and the Beck Depression Inventory-II (α = 0.87; sample item: “My future seemed dark to me”; Beck et al., 1974, 1996). The results of a confirmatory factor analysis estimating the four first-order factor and the second-order factor of mental health issues confirmed the scale's theoretical structure [χ^2^(163) = 986.21, *p* < 0.001; CFI = 0.915; TLI = 0.901; RMSEA = 0.071; SRMR = 0.049]. Thus, scores of mental health issues were computed as the mean of the 20 items and showed excellent internal consistency (α = 0.93).

#### Offline and Online Social Contacts

Quantity and quality of social contacts were measured separately for offline (i.e., face-to-face) and online (i.e., mediated by phone and social media) contacts, using a listing procedure adopted in previous research (Page-Gould, 2012; Marinucci and Riva, 2020b). Specifically, participants were asked to list up to 10 persons they had interacted with during the previous week and rate how close they felt to each of them on a Likert scale ranging from 1 (not close at all) to 5 (extremely close). A final check question was included for both offline and online contacts. Specifically, if participants did not fill in any list entry, they were asked if it was intended (meaning that they had 0 offline or online contacts) or if it was a mistake. In the latter case, participants were asked to go back and fill in the list. Scores were computed as the sum of closeness rates for each person reported, obtaining two separate indices for offline and online contacts, respectively. Based on the check questions, scores of 0 were given to participants who left the list blank on purpose. Offline and online social contacts were randomly presented to the participants to control for possible order effect.

#### Space Adequacy

Three items were developed *ad hoc* to measure the adequacy of the space where participants were currently living. Participants were asked to rate how adequate were the (1) size, (2) brightness, and (3) privacy of their living space on a Likert scale ranging from 1 (not at all) to 7 (extremely). The space adequacy score was computed as the mean of the three items and showed adequate internal consistency (α = 0.73).

## Results

### Preliminary Analysis

The regional percentage of COVID-19-positive cases over the total number of infected in the Italian population ranged between 0.23 and 58.28%, *M* = 31.86, SD = 23.20. According to the regional severity of contagion, the sample was split into a “low contagion” (LC) subsample (*n* = 414), range: 0.23–13.94%, *M* = 4.73, SD = 4.75, and a “high contagion” (HC) subsample (*n* = 592), range: 43.71–58.28%, *M* = 50.83, SD = 4.85. Descriptive statistics of and comparison between the two subsamples on sociodemographic characteristics and predictor variables are presented in Table 1. All the variables did not differ between the two subsamples, except the number of people living with participants and days of forced social isolation. Specifically, compared with LC, HC participants lived with more people and completed the survey almost 1 day before, on average. However, these differences were associated with small effect sizes; thus, they were considered negligible.

**Table 1:** Descriptive statistics of and comparison between the low- and high-contagion subsamples.

	**Low contagion (*n* = 414)**	**High contagion (*n* = 592)**	**Inferential statistic**	**Effect size**
Gender			χ^2^(1) = 3.19, *p* = 0.074	ϕ = 0.056
Males	93 (22.5%)	106 (17.9%)		
Females	321 (77.5%)	486 (82.1%)		
Age	29.55 (10.82)	29.58 (10.95)	*t*(1004) = 0.05, *p* = 0.959	*d* = 0.003
Nationality			χ^2^(1) = 0.00, *p* = 0.981	ϕ = 0.001
Italian	405 (97.8%)	579 (97.8%)		
Other	9 (2.2%)	13 (2.2%)		
Occupation			χ^2^(1) = 0.01, *p* = 0.931	ϕ = 0.003
Employed	240 (58.0%)	341 (57.6%)		
Not employed	174 (42.0%)	250 (42.2%)		
Education			χ^2^(2) = 1.84, *p* = 0.400	ϕ = 0.043
<High school	17 (4.1%)	18 (3.0%)		
High school	197 (47.6%)	266 (44.9%)		
≥Bachelor	200 (48.3%)	308 (52.0%)		
People living with participants	2.10 (1.32)	2.31 (1.30)	*t*(1002) = 2.47, *p* = 0.014	*d* = 0.158
Social isolation	10.57 (4.90)	9.73 (4.64)	*t*(1004) = 3.01, *p* = 0.003	*d* = 0.193
Space adequacy	4.84 (1.43)	4.95 (1.38)	*t*(1004) = 1.23, *p* = 0.218	*d* = 0.079

*Number of cases and percentages within the subsample (in brackets) were reported for categorical variables (i.e., gender, nationality, occupation, education), mean (and SD) for the other variables*.

### Main Analysis

**Figure 1:**
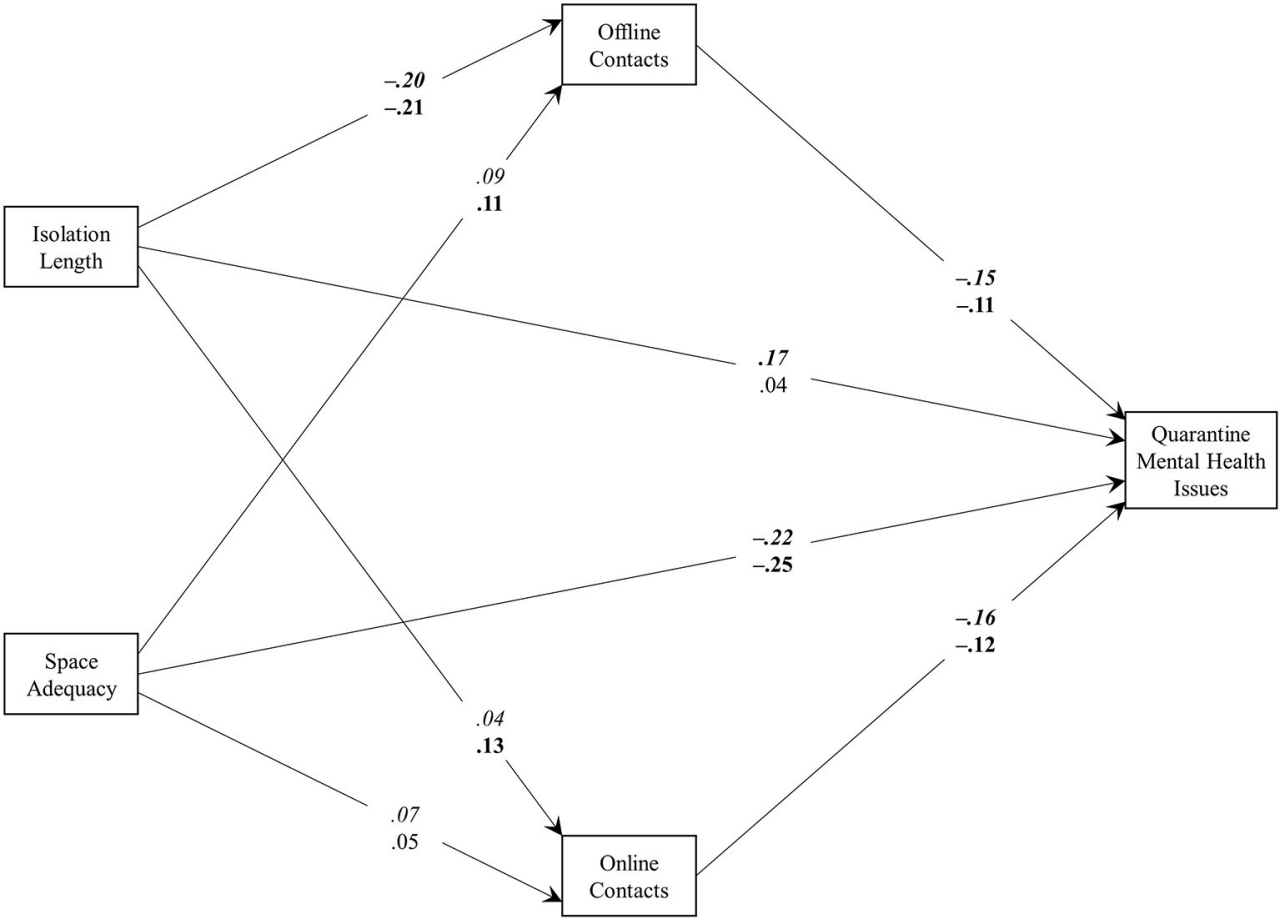
The results of the multi-group path analysis model. Parameters for the low-contagion and high-contagion subsamples are reported in italics and plain text, respectively. Parameters in bold are significant at level *p* < 0.05.

A multi-group path analysis investigated the association of social isolation length and space adequacy with mental health issues and whether these relationships were mediated by offline and online contacts, estimating separate models for participants in low-contagion (LC; *n* = 414) and high-contagion (HC; *n* = 592) areas. Gender and age were entered as further predictors of the two mediators and the outcome to control for their effect. The analysis was run using Mplus, version 7 (Muthén and Muthén, 2012). The model is graphically depicted in Figure 1; the complete list of parameters is reported in Table 2. The variance of mental health issues explained by the model was 0.23 for LC and 0.14 for HC.

**Table 2:** The results of the multi-group path analysis: standardized regression coefficients and 95% CIs are reported.

	**Low contagion (*****n*** **=** **414)**			**High contagion (*****n*** **=** **592)**		
	**coeff**	**95% CI**	***p***	**coeff**	**95% CI**	***p***
**Regressions**						
Isolation length → MHI	0.17	0.08, 0.26	<0.001	0.04	−0.04, 0.12	0.278
Space adequacy → MHI	−0.22	−0.31, −0.14	<0.001	−0.25	−0.32, −0.18	<0.001
Offline contacts → MHI	−0.15	−0.24, −0.06	0.001	−0.11	−0.19, −0.03	0.005
Online contacts → MHI	−0.16	−0.24, −0.07	<0.001	−0.12	−0.20, −0.05	0.001
Gender → MHI	0.01	−0.08, 0.09	0.860	−0.05	−0.13, 0.02	0.181
Age → MHI	−0.20	−0.29, −0.12	<0.001	−0.13	−0.21, −0.06	0.001
Isolation length → offline contacts	−0.20	−0.29, −0.10	<0.001	−0.21	−0.28, −0.13	<0.001
Space adequacy → offline contacts	0.09	−0.00, 0.18	0.059	0.11	0.03, 0.19	0.006
Gender → offline contacts	0.12	−0.03, 0.22	0.010	0.06	−0.02, 0.14	0.143
Age → offline contacts	0.03	−0.06, 0.13	0.493	−0.03	−0.11, 0.05	0.514
Isolation length → online contacts	0.04	−0.05, 0.14	0.360	0.13	0.05, 0.21	0.001
Space adequacy → online contacts	0.07	−0.03, 0.16	0.155	0.05	−0.03, 0.13	0.195
Gender → online contacts	−0.08	−0.17, 0.02	0.103	−0.04	−0.12, 0.04	0.377
Age → online contacts	0.19	0.10, 0.29	<0.001	0.07	−0.02, 0.15	0.109
**Correlations**						
Offline contacts–online contacts	0.16	0.06, 0.25	0.001	0.12	0.04, 0.20	0.002
**Intercepts**						
MHI	4.29	3.85, 4.73	<0.001	4.60	4.23, 4.97	<0.001
Offline contacts	1.60	1.13, 2.07	<0.001	1.67	1.29, 2.06	<0.001
Online contacts	1.30	0.81, 1.79	<0.001	1.48	1.08, 1.88	<0.001
**Residual variances**						
MHI	0.77	0.70, 0.84	<0.001	0.86	0.81, 0.91	<0.001
Offline contacts	0.93	0.88, 0.98	<0.001	0.95	0.91, 0.98	<0.001
Online contacts	0.95	0.91, 0.99	<0.001	0.97	0.95, 1.00	<0.001

*All the parameters were standardized. The column “coeff.” reports β for regressions and Pearson r for correlations. Gender was coded as 0 for females and as 1 for males. MHI, mental health issues*.

While space adequacy was negatively associated with mental health issues for both LC and HC participants, social isolation had a significant, direct effect on it only for the LC subsample: the longer the isolation, the higher the mental health issues. Moreover, the direct effect of space adequacy on mental health issues was significantly higher than that of isolation length for both LC, Δb = 0.23, *p* < 0.001, and HC, Δb = 0.21, *p* < 0.001. Both offline and online social contacts had significant negative associations with mental health issues, irrespectively from the level of contagion. For both LC and HC participants, (a) the longer the social isolation, the less the offline contacts, and (b) the worse the space adequacy, the fewer the offline contacts. Conversely, the only significant association of online contacts was with the length of isolation, which showed a positive effect only for the HC subsample.

Mediation paths were evaluated using the bootstrapping technique, computing the 95% CI based on 5,000 resamplings. Concerning the LC subsample, offline contacts significantly mediated the relationship between social isolation and the outcome, β = 0.029, 95% CI [0.006, 0.051], meaning that the negative association between forced isolation and mental health was partially due to reduced face-to-face contacts. The total effect (i.e., direct plus indirect effects) of the length of isolation on mental health issues was significant and positive, β = 0.191, 95% CI [0.108, 0.275]. Conversely, offline contacts did not mediate the link between space adequacy and mental health issues, β = −0.013, 95% CI [−0.029, 0.002], and no indirect effects through online contacts were found (social isolation: β = −0.007, 95% CI [−0.022, 0.008]; space adequacy: β = −0.011, 95% CI [−0.027, 0.005]).

Concerning the HC subsample, both offline, β = 0.023, 95% CI [0.005, 0.041], and online, β = −0.016, 95% CI [−0.031, −0.001], contacts were significant mediators of the relationship between social isolation and the outcome. This means that, if fewer and less satisfying face-to-face contacts might decrease mental health due to forced isolation, the quantity and quality of online contacts might buffer the negative consequences of social isolation. The total effect (i.e., direct plus indirect effects) of the length of isolation on mental health issues was not significant, β = 0.049, 95% CI [−0.023, 0.122]. This was likely due to the two mediation effects that had an opposite sign. Offline contacts, β = −0.012, 95% CI [−0.024, 0.000], and online ones, β = −0.007, 95% CI [−0.018, 0.005], did not significantly mediate the link between space adequacy and mental health issues.

## Discussion

The COVID-19 pandemic led many nations to impose severe social restrictions on their citizens. Although this measure contained the virus spread, it could also have significant repercussions on people's mental health. However, different lockdowns have had different characteristics. In Italy, to date, no lockdown has had the level of severity of the one imposed in March and April 2020.

Based on 1,006 respondents in Italy, our data showed that forced isolation due to the first wave of COVID-19 could be associated with lower mental health constructs typically considered rather stable, especially in areas experiencing relatively low levels of contagion. Although the research design did not allow making causal inferences, the present findings indicated that, even in a relatively brief time span, social deprivation could lead to relevant repercussions for individuals' psychological well-being, showing that the longer the isolation, the worse the mental health.

Previous knowledge of social isolation's effects could be considered with what is revealed by our data according to two main standpoints. First, the COVID-19, as an exceptionally extreme event in recent history, may have elicited intense feelings of fear and threat for human survival, boosting the development of psychological issues. This result is consistent with studies that linked the pandemic with increased feelings of anxiety and depression (e.g., Hervalejo et al., 2020; Roma et al., 2020; Torales et al., 2020). Second, the virus local spread could have further moderated the impact of imposed social isolation on mental health. In this fashion, our findings add to the literature linking the virus local spread to its repercussions on mental health, helping to clarify the mixed results obtained so far (Ahmed et al., 2020; Gao et al., 2020). Accordingly, people in low-contagion areas might perceive the government's restrictions as exaggerated for their current situation and would suffer more from forced isolation. Conversely, people in high-contagion areas might better understand the need for physical distancing, accepting it, and perceiving the adherence to it as essential.

In high-contagion areas, we found a positive association between isolation length and online social contacts. Likely, the restrictions to face-to-face contacts could have led people to seek more online connections, which seemed to buffer against social restrictions' negative impact. This did not occur in low-contagion areas. This finding concurs with the aforementioned speculation in explaining the direct and stronger relationship between the length of isolation and mental health in low-contagion areas. Thus, it is possible that, compared with participants in low-contagion areas, the higher perception of threat could have prompted those in high-contagion areas to search (and, eventually, provide) social support through online contacts, protecting them from the adverse effect of isolation length. This interpretation is consistent with studies on the beneficial impact of online social contacts in disadvantaged individuals (e.g., physically restricted elderly; Delello and McWhorter, 2015) and people facing health challenges (e.g., breast cancer patients; Fogel et al., 2002), indicating that previous results might be broadened to the general population. Moreover, our results enrich the ongoing debate on social networking sites' role on well-being, which is at least controversial and primarily focused on their negative effects (Kuss and Griffiths, 2017). Several authors highlighted the risks of screen time—also encompassing social networking—for mental health, given that the time spent online reduced the commitment to offline activities and interactions (Twenge et al., 2018). Conversely, the present results seem to indicate that online social connections can replace the supportive effect of face-to-face interactions, especially when the latter are not available and in times of uncertainty and mass threat (in line with Waytz and Gray, 2018).

Crucially, we also found a key role in space adequacy in both low- and high-contagion areas. Indeed, the more adequate the space where participants were confined, the fewer mental health issues. This finding is consistent with both studies suggesting that inadequate living space can compromise psychological well-being (e.g., WHO/Europe, 2007; Jones-Rounds et al., 2014). Moreover, this association was even stronger than the one between the length of isolation and mental health. This result underlines the role of economic inequalities in relation to people's psychological well-being, suggesting that people who have the opportunity to live in relatively large and bright houses, guaranteeing privacy, suffer significantly less from the adverse effects of lockdown. Inevitably, special attention should be devoted when considering the impact of forced isolation for those living in inadequate dwellings.

### Limitations and Future Research

There are some limitations regarding the current study. A first limitation is related to the constructs considered in this study: we cannot exclude that third variables (e.g., increased fear of infection or the exposure to information on the pandemic considered, as in Gao et al., 2020) may account for the effects we have found. Importantly, the assessment of participants' mental illnesses would have been a useful control variable to exclude confounding effects deriving from pre-existing conditions. Similarly, our measure of space adequacy is not comprehensive of all the features that might be relevant in a condition of self-isolation at home, such as the availability of outdoor spaces (Soga et al., 2021).

A second limitation is related to our sample. Indeed, the prevalence of women and the slightly different composition of the two subsamples (i.e., LC and HC) in terms of number of people living with the participants and length of social isolation limited our results' generalizability, suggesting the need for future studies.

However, the main limitation concerns its cross-sectional design. Although we have followed the evolution of the pandemic's most critical phase over multiple days, the lack of baseline social contacts and mental health measures before the COVID-19 outbreak limited the interpretation of relationships among variables in a causal fashion. Nonetheless, the rationale of the model tested is supported by some theoretical considerations. First, the lockdown length and the virus regional spread, as objective indicators, could not depend on self-report measures and were reasonably treated as exogenous predictors. Second, the literature on social disconnection has demonstrated the detrimental effects of physical and emotional separation from others on well-being (Baumeister and Leary, 1995; Smart Richman and Leary, 2009; Williams, 2009; Holt-Lunstad et al., 2010; Riva and Eck, 2016), supporting the inclusion of offline and online contacts as predictors of mental health issues in our model. Third, although space adequacy was not an objective indicator, it was still included among the exogenous predictors, consistently with the literature that links the characteristics of the living space with the experience of isolation and well-being (WHO/Europe, 2007; Jones-Rounds et al., 2014). However, as a self-report measure, we must acknowledge a possible bi-directional influence between space adequacy and mental health. In other words, as people living in less satisfying physical spaces might suffer more from the lockdown, people suffering more from the lockdown might also have a worse perception of the living space where they are confined, which might be partially independent of the objective characteristics of the physical space itself. Again, this is a limitation that future studies might address through longitudinal design that links the length of isolation with mental health issues during a lockdown. Such studies should also consider the spread of contagion in the residence area and varying degrees of face-to-face and online connections.

### Conclusions and Practical Implications

Beyond the advancement of the psychological impact of the COVID-19 lockdown, the exceptional nature of the COVID-19 pandemic made it possible to study an objective form of social isolation on a large scale, instead of limiting the research on certain marginalized social groups (e.g., Aureli et al., 2020). Moreover, assessing the level of contagion based on official, objective reports related to the participants' geographical area and the exact day of survey completion can be considered a strength of the present study.

Findings related to the local contagion rate's moderating role suggest governments and policymakers take special care to explain the reasons for forced isolation in areas where the infection rate is low. Moreover, the present results indirectly support the tier-based restrictive measures currently adopted by many governments. Indeed, adjusting the lockdown restrictions according to the local contagion rate might be crucial to prevent (or, at least, reduce) mental health issues, especially for people living in areas where the COVID-19 spread is limited. Nevertheless, our data do not allow a direct test of these speculations; thus, future studies should investigate further the plausibility of our interpretations.

Overall, this study suggests that restricting people's mobility, although essential to slow the spread of the infection, can put a significant strain on people's mental health on a scale unprecedented in recent history. Thus, in addition to trying to slow the spread of the pandemic, we must work to make multiple forms of psychological support available to manage the most critical situations, that is, those who have (a) few face-to-face contacts, (b) limited ability to use online contacts as buffers, and (c) inadequate physical spaces to live in.

## Data Availability Statement

The dataset presented in this study can be found in the OSF repository at the following link: https://osf.io/xb8yj/?view_only=966abafccc844b99924da85be3f76272.

## Ethics Statement

The study involving human participants was reviewed and approved by Ethics Committee of the Department of Psychology at the University of Milan—Bicocca. The participants provided their informed consent to participate in this study.

## Author Contributions

LP conducted the data analysis. PR supervised the research project. All the authors contributed equally to the conception and the design of the work, as well as to data collection, writing the first draft of the article and interpreting results, agreed to all aspects of the work, and approved the version to be published.

## Conflict of Interest

The authors declare that the research was conducted in the absence of any commercial or financial relationships that could be construed as a potential conflict of interest.
